# Pseudomonas aeruginosa bloodstream infections in internal medicine wards: A large Italian multicenter retrospective study

**DOI:** 10.1371/journal.pone.0317540

**Published:** 2025-05-19

**Authors:** Silvia Corcione, Simone Mornese Pinna, Antonio Vena, Michela Schenone, Maura Sanino, Renato Pascale, Emanuele Pivetta, Daniele Roberto Giacobbe, Maddalena Giannella, Nour Shbaklo, Francesca Giovannenze, Nicholas Geremia, Malgorzata Mikulska, Davide Bavaro, Eleonora Taddei, Vincenzo Scaglione, Veronica Vassia, Benedetta Fumarola, Michele Bartoletti, Pierluigi Viale, Matteo Bassetti, Francesco Giuseppe De Rosa, on behalf of SITA GIOVANI (Young Investigators Group of the Società Italiana Terapia Antinfettiva)

**Affiliations:** 1 Department of Medical Sciences, Infectious Diseases, University of Turin, Turin, Italy; 2 Tufts University School of Medicine, Boston, MA, United States of America; 3 Department of Health Sciences (DISSAL), University of Genoa, Genoa, Italy; 4 Clinica Malattie Infettive, IRCCS San Martino polyclinic Hospital, Genoa, Italy; 5 Department of Medical Sciences, Internal Medicine, University of Turin, Turin, Italy; 6 Infectious Diseases Unit, IRCCS—Sant’Orsola Polyclinic, Bologna, Italy; 7 Department of Medical and Surgical Sciences, University of Bologna, Bologna, Italy; 8 Department of Laboratory and Infectious Sciences, IRCCS A. Gemelli University polyclinic Foundation, Rome, Italy; 9 Unit of Infectious Diseases, Department of Clinical Medicine, Dell’Angelo Hospital, Venice, Italy; 10 Unit of Infectious Diseases, Department of Clinical Medicine, Ospedale Civile S.S. Giovanni e Paolo, Venice, Italy; 11 Department of Biomedical Sciences, Humanitas University, Milan, Italy; 12 Infectious Disease Unit, IRCCS Humanitas Research Hospital, Rozzano, Milan, Italy; 13 Infectious and Tropical Diseases Unit, Padua University Hospital, Padua, Italy; 14 Infectious and Tropical Disease Unit, Mauriziano Umberto I Hospital, Turin, Italy; 15 Infectious and Tropical Disease Unit, Civile Hospital, Ivrea, Italy; 16 Infectious Disesases, Ospedali Civili, Brescia, Italy; ARNAS Civico Di Cristina Benfratelli: Azienda Ospedaliera di Rilievo Nazionale e di Alta Specializzazione Civico Di Cristina Benfratelli, ITALY

## Abstract

**Introduction:**

This large, multicenter, Italian retrospective study aimed to describe clinical characteristics and risk factors associated with 30-day mortality in patients with Pseudomonas aeruginosa bloodstream infections (PA-BSI) admitted to internal medicine wards (IMW). To enhance clinical decision-making, we also developed and internally validated a bedside prognostic model to predict the 30-day mortality risk.

**Methods:**

We conducted a retrospective, multicenter cohort study across 14 public hospitals in Italy, analyzing all adult patients admitted to IMW with PA-BSI between 2021 and 2022.

**Results:**

Out of 285 eligible patients with PA-BSI, the median age was 73 years, and septic shock occurred in 13.3% of cases. Less than 5% of PA-BSI were caused by difficult-to-treat resistant P. aeruginosa (DTR-PA). Encouragingly, appropriate empiric therapy was administered in 69.8% of patients, yet the overall 30-day mortality remained 22.5%. Cox regression analysis identified age, and urinary catheter use as significant risk factors for mortality. Conversely, adequate source control and targeted therapy emerged as protective factors. Multivariate analysis confirmed septic shock at bloodstream infection onset (HR 6.96; 95% CI, 1.72–28.12) as a strong independent predictor of mortality, whereas effective source control (HR 0.152; 95% CI, 0.039–0.59) significantly improved survival odds. Using these insights, we developed a practical prognostic model capable of estimating the 30-day mortality risk, providing clinicians with a valuable predictive tool at the bedside.

**Conclusions:**

PA-BSI in IMWs is characterized by a relatively low incidence of septic shock and rate of resistance, alongside high rates of appropriate empiric therapy. Despite these favorable factors, meropenem may represent a valuable therapeutic option for PA-BSI with severe presentations in this setting since mortality remains substantial (22.5%). Our findings underscore the critical importance of early source control and identify septic shock as a key predictor of mortality even in the setting of IMW. The proposed bedside nomogram model could empower clinicians to identify high-risk patients early, facilitating timely interventions and improving outcomes in this vulnerable population.

## Introduction

*Pseudomonas aeruginosa* (PA) is a leading cause of bloodstream infections (BSI) and is associated with high attributable mortality [1^–^5]. Most studies have focused on the characteristics and management of the disease in onco-hematological or critically ill patients. However, in recent years, attention has shifted towards episodes of PA-BSI occurring in surgical wards or in post-surgicalpatients [1–3]. The success of PA as a pathogen can be attributed to its complex genomic and metabolic adaptations. These include various innate defense mechanisms, a wide range of virulence genes, the ability to form biofilms, and a remarkable capacity to develop and disseminate antimicrobial resistance in host organisms [4]. In 2018, a new classification for pathogens resistant to all primary treatment options, termed Difficult-to-Treat Resistance (DTR) has been introduced by Infectious Diseases Society of America (IDSA), defined as resistance to fluoroquinolones and all antipseudomonal β-lactams, including carbapenems, regardless of susceptibility to β-lactam/β-lactamase inhibitor (BL-BLI) and aminoglycosides [5]. The introduction of novel antibiotics (ceftolozane/tazobactam, ceftazidime/avibactam imipenem/relebactam, cefiderocol) has changed the therapeutic scenario in the setting of DTR-PA [6]. While there is extensive knowledge about antibiotic resistance patterns, clinical manifestations, and potential outcomes in patients with severe preexisting conditions, critically ill and immunocompromised hosts, the burden and specific risk factors for mortality in patients with PA-BSI admitted to internal medicine wards (IMW) have not been thoroughly investigated.

The aim of the study was to assess clinical characteristics and risk factors for 30-day mortality among patients with PA-BSI admitted to IMW. The secondary aim was to provide a prognostic tool to predict 30 days mortality in IMW patients with PA-BSI.

## Materials and methods

### Study setting and data collection

This retrospective multicenter observational study included patients with BSI due to PA occurring in hospitalized patients admitted to IMW or a subspecialty ward of internal medicine at the time their positive blood cultures were drawn, between January 1^st^ 2021 and December 31^st^ 2022. Data analyzed in this study were derived from the dataset “OPERA Observership Project 2023”; part of this cohort has been published elsewhere [7]. This dataset served as the primary source for our current analysis, allowing us to explore additional research questions and expand upon the findings of the previous study

The study was carried out in 14 hospitals located in 8 Italian regions (Liguria, Piedmont, Lombardy, Lazio, Veneto, Emilia-Romagna, Apulia, and Sicily) through the SITA GIOVANI network (Observership Project 2023].

In all centres, an infectious disease specialist tracked patients with BSI, from the time of positive blood cultures until the outcome (i.e., death, discharge, or up to 30 days post-diagnosis, whichever occurs first).

### Definitions

Hospitalized patients with positive blood cultures for PA were considered in the study if they were aged ≥ 18 years, received at least 24 hours of antibiotic treatment and had a culture-confirmed PA-BSI during IMW stay. Patients were excluded if death was registered within the first 24 hours after BSI detection to reduce selection bias.

According to our study protocol, antibiotic treatment was defined appropriate when at least one antibiotic with antimicrobial activity against PA resulted active *in vitro*.

Appropriate empiric therapy was defined as the administration of at least one active *in vitro* agent against PA within 48h from culture collection. Targeted therapy was defined as the administration of at least one active *in vitro* agent against PA after blood culture susceptibility results. Colistin and aminoglycoside monotherapy were considered inappropriate as empiric treatment for non-urinary sources of BSI.

Antibiotics were classified as:

1) antipseudomonal β-lactams (i.e., ceftazidime, cefepime, piperacillin/tazobactam);
2) new antipseudomonal β-lactams (i.e., ceftolozane/tazobactam, ceftazidime/avibactam, meropenem/vaborbactam, imipenem/cilastatin, relebactam);
3) antipseudomonal carbapenem (i.e., meropenem, imipenem).

### Collection of epidemiologic data

Medical charts were retrospectively reviewed including the following variables: age, gender and clinical comorbidities (including cardiovascular disease, heart failure, diabetes, chronic renal failure, patient on dialysis, hepatopathy, neurological disease, previous therapy with steroid or other immunosuppressive drugs, previous splenectomy, previous antibiotic therapy regimen, surgery or endoscopic procedures, presence of invasive devices, Charlson Comorbidity Index score, previous hospitalization within the previous 90 days). Neutropenia was defined as an absolute neutrophil count of less than 500 cells/μL at the time of bloodstream infection onset. Immunosuppression was defined as: recipient of solid-organ transplant, long-term (≥ 28 days), use of corticosteroids (prednisone >25 mg/day or equivalent corticosteroid for more than 7 days) or other immunosuppressant drugs, human immunodeficiency virus (HIV) infection, and genetic causes of immunodeficiency. Immune status was recorded at the time of collection of blood cultures. Haematological and solid organ malignancies were defined as “cancer”. Invasive procedures as placement of urinary catheter, central venous catheter (CVC) or surgery were recorded either during hospitalization or within 30 days prior to the onset of PA-BSI. Infection related characteristics of PA-BSI events included prior colonization and antimicrobial susceptibility of PA within the previous 90 days; the origin of the infection and the source control; antibiotic therapy against PA and outcomes including overall 30-day mortality.

Blood cultures, organism identification, and susceptibility testing were conducted at each participating centre based on their standard operating procedures. Antibiotic susceptibilities were defined according to the guidelines of the European Committee on Antimicrobial Susceptibility Testing (EUCAST v 13.0) [8].

### Other definitions

An episode of PA*-*BSI was defined as the presence of P. *aeruginosa* in at least one blood culture sample, either from central or peripheral blood. BSI onset was defined as the date of collection of the first blood culture yielding the study isolate. Septic shock was recorded on the first 24 hours of PA-BSI onset and it was defined in line with the third international consensus definition for sepsis and septic shock (Sepsis-3) [9]. As for the source of BSI, diagnosis and classification of infection were defined according to the criteria of the US Centers for Disease Control and Prevention (CDC) [10]. Adequate source control was defined as the removal of pre-existing infected hardware or drainage of an infected fluid collection thought to be the origin of the PA-BSI, performed within 24 hours from the BSI onset.

An episode of catheter-related BSI (CRBSI) was diagnoses when PA grew from a peripheral blood drain and from a culture of the catheter tip, or when peripheral vein and central line cultures fulfilled quantitative CRBSI criteria or differential time to positivity (DTP) [11].

Probable origin of BSI was defined as “primary” when the source of the infection was unclear.

The presence of coagulase-negative staphylococci (CoNS) from single blood culture in which PA grew was not considered as “polymicrobial” but rather as a contamination from a normal skin-resident organism.

### PA isolates were classified as follow, according to current definitions

- Susceptible (MS): when strains were inhibited by a standard concentration of antimicrobial agents active against P. *aeruginosa;*
- Multi drug resistant (MDR) PA: the isolate is non-susceptible to at least 1 agent in 3 antimicrobial categories among antipseudomonal β-lactams (including ceftazidime, cefepime, piperacillin/tazobactam) meropenem, monobactams, aminoglycosides, fluoroquinolones, fosfomycin, and polymyxins;
- difficult-to-treat resistance (DTR) PA: the isolate exhibited non-susceptibility to antipseudomonal fluoroquinolones, piperacillin-tazobactam, antipseudomonal cephalosporins, aztreonam, meropenem, imipenem-cilastatin [12].

### Endpoints

The primary endpoint of the study was to assess clinical characteristics and risk factors for 30-day mortality among patients with PA BSI admitted to IMW. The secondary aim was to provide a prognostic tool to predict mortality in IMW patients with PA BSI.

### Ethics

The study was approved by the institutional review board of the coordinating centre (Città della Salute e della Scienza di Torino University Hospital, Turin, Italy, N. 202/2023, PROT.N. 0066633) and was in accordance with the declaration of Helsinki. The need for informed consent was waived by Ethics committee. All data was fully anonymized before being accessed for analysis by researchers. Data were accessed for research purposes from May 1^st^ 2023.

### Statistical analysis

All data was collected using REDcap platform.

Demographic and clinical characteristics of enrolled patient were reported as median and interquartile range or mean and standard deviation for continuous data or number and percentage for ordinal data, as appropriate.

Multiple logistic models were performed to test the potential causal effect of several covariates on the outcome. Multivariate analysis was performed using the clinically significative variables identified.

Kaplan-Meier method was used to estimate 30-day mortality. Time to event was recorded while non-events were censored. Cox proportional hazard analyses were used to model the effects of covariates on mortality.

In order to provide a prognostic model for predicting mortality in patients with PA BSI based on their Spearman rho2 score and the previous use in the international literature, prognostic factors were identified based on potential covariates explored during the study that resulted significantly associated with the primary outcome and demonstrated clinical relevance. The discrimination ability was assessed using the C-index [13]. The calibration was visually assessed by evaluating the plot of the observed vs. predictive probability of death after a 5000-replication bootstrapping procedure [14]. A nomogram, a graphical presentation of such a model, was proposed as an easy-to-use tool for applying the prognostic model. A 2-side *p* value <0.05 was considered significative.

Analyses were performed using STATA 18 SE (Stata Corp, TX, College Station, TX, USA) and R 3.6.3 (The R Foundation for Statistical Computing, 2020].

## Results

### Patients

During the 2-year study period, a total of 639 episodes of PA-BSI were identified. Of these, 183 (28%) were in the ICU, 71 (11%) in hematology wards, 100 in surgical wards (15%). The remaining 285 cases (44.6%) occurred in adult patients admitted to IMW and are the subject of the present study (**Table 1**).

**Table 1:** Demographics and Clinical Characteristics of Patients with *P. aeruginosa* BSI, Categorized by 30 days mortality.

Variable	Overall (n = 285)	Alive (n = 221; 77.5%)	Death (n = 64; 22.5%)	*P* value
Age, (yr)	73 (18-94)	70 (18-94)	78 (39-92)	0.0003
Charlson score	4 (0-15)	4 (0-15)	5 (0-13)	0.183
Sex (male)	187 (65.6%)	139 (62.8%)	40 (62.5%)	0.551
Comorbidities				
Diabetes	68 (23.2%)	52 (23.5%)	16 (25.0%)	0.973
Neurological disease	105 (36.8%)	79 (35.7%)	26 (40.6%)	0.476
Cardiovascular disease	100 (35.1%)	75 (33.9%)	25 (39.1%)	0.449
COPD	41 (14.4%)	29 (13.1%)	12 (18.7%)	0.259
Renal failure	62 (21.6%)	51 (23.1%)	11 (17.2%)	0.315
Renal failure on dyalisis	19 (6.7%)	15 (6.8%)	4 (6.3%)	0.571
Cirrhosis	9 (3.2%)	0 (0.0%)	9 (4.1%)	0.098
Cancer	61 (21.4%)	43 (19.5%)	18 (28.1%)	0.137
Severe neutropenia (<500 neutrophil/uL)	21 (7.4%)	13 (5.9%)	8 (12.5%)	0.074
Septic shock at time of BSI	38 (13.3%)	18 (8.1%)	20 (31.2%)	<0.01
Central venous line	143 (50.2%)	113 (51.1%)	30 (46.9%)	0.549
Urinary catheter	168 (58.9%)	121 (54.8%)	47 (73.4%)	0.007
Corticosteroids and iatrogenic immunosuppression	23 (8.1%)	20 (9.1%)	3 (4.7%)	0.259
Chemotherapy	26 (9.1%)	17 (7.7%)	9 (14.1%)	0.119
Source of infection				
UTI Pneumonia Primary BSI CRBSI Other	47 (16.5%) 24 (8.4%) 104 (36.5%) 58 (20.3%) 52 (18.2%)	42 (19.0%) 15 (6.8%) 69 (31.2%) 52 (23.5%) 43 (19.5%)	5 (7.8%) 9 (14.1%) 35 (54.7%) 6 (9.4%) 9 (14.1%)	0.052 0.023 0.070 0.015 0.557
Adequate source control	81 (28.4%)	77 (34.8%)	4 (6.3%)	0.001
Empiric therapy	227 (79.6%)	169 (76.5%)	58 (90.6%)	0.562
Empiric appropriate therapy	199 (69.8%)	156 (70.6%)	43 (67.2%)	0.230
Target only therapy	39 (13.7%)	33 (14.9%)	6 (9.3%)	0.009
DTR-PA	14 (4.9%)	11 (4.9%)	3 (4.7%)	0.766

Continuous variables are described as median and interquantile range (IQR), whereas categorical variables are represented as frequencies and percentages.

The median age was 73.0 (range, 18.0–94.0) and 65.6% were males. Median Charlson score at the time of BSI onset was 4 (range 0–15). Neurological diseases (105, 36.8%), cardiovascular diseases (100, 35.1%), diabetes (68, 23.2%), chronic kidney failure (62, 21.6%) and chronic obstructive pulmonary disease (41; 14.4%) were the most common underlying conditions. Of the 285 patients, 26 (9.1%) had chemotherapy and 23 (8.1%) were on immunosuppressive agents or corticosteroid treatment.

At the time of presentation of PA-BSI, 143 (50.2%) patients had a central venous line, and 168 (58.9%) had a urinary catheter. Septic shock was observed in 38 (13.3%) patients.

### Source Of infection and microbiology

The origin of PA-BSI was central-venous line in 58 (20.3%), urinary tract in 47 (16.5%), and low respiratory tract in 24 (8.4%). In 104 (36.5%) patients, BSI was defined as “primary” as a known source of infection was not found and criteria for CRBSI were not fulfilled. Of the 285 isolated strains, 14 (4.9%) were classified as DTR-PA.

### Antibiotic treatment and source control

Two hundred twenty-seven (79.6%) patients were treated with empiric therapy; 199 (69.8%) was appropriate and 28 (12.3%) inappropriate. Among patients who received appropriate empiric treatment, 163 (81.9%) received a monotherapy, and 36 (18.1%) received a combination treatment. Among patients who received empirical treatment, 112 (49.3%) received antipseudomonal β-lactams and 60 (26.4%) carbapenems; 39(13.7%) patients did not receive any empiric treatment but received target-only therapy (ToT) administered after blood culture results. Source control was performed in 81 (28.4%) patients.

### Outcomes: 30- days mortality and risk factors for mortality

**Fig 1:**
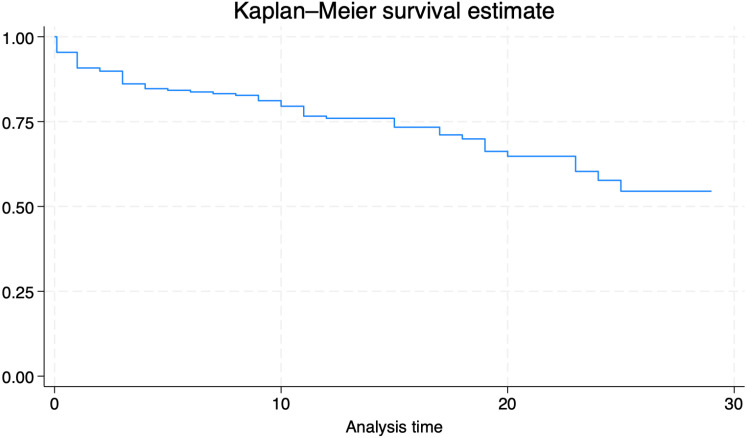
Kaplan MEIER curve of overall survival in our cohort.

Overall, 30-day mortality was 22.5% (64 deaths); the overall survival is described in **Fig. 1**. At cox-regression analysis age and urinary catheter were identified as factors significantly associated with 30-day mortality, whilst adequate source control and TOT were protective (**Table 2**). In the multivariate model, adequate source control (HR, 0.152 [95% CI, 0.039–0.59]) remained significant, while septic shock at time of BSI (OR, 6.962 [95% CI, 1.72–28.12]) was an independent risk factor for 30-day mortality (**Table 3**).

**Table 2:** Cox regression for overall 30-day mortality.

Variable	HR	P value	IC 95%
Age	1.039	**0.001**	**1.01-1.062**
Neutropenia	1.27	0.251	0.73-3.24
Steroids and iatrogenic immunosuppression	1.4	0.062	0.82-2.4
Adequate source control	0.11	**0.000**	**0.368-0.38**
Urinary catheter	2.32	**0.003**	**1.33-4.06**
Target only therapy (ToT)	0.24	**0.021**	**0.07-0.81**

**Table 3:** Multivariate analysis for 30-day mortality.

Variable	HR	Std.err	p	IC 95%
Age	0.679	0.575	0.647	0.13-3.568
Neutropenia	1.447	1.623	0.742	0.161-13.036
Immunesuppression	0.581	0.472	0.504	0.12-2.86
**Adequate source control**	**0.152**	**0.105**	**0.006**	**0.039-0.59**
**Septic shock**	**6.962**	**4.960**	**0.006**	**1.72-28.12**

### Nomogram as a bed side tool to predict mortality in PA-BSI in IMW

**Fig 2:**
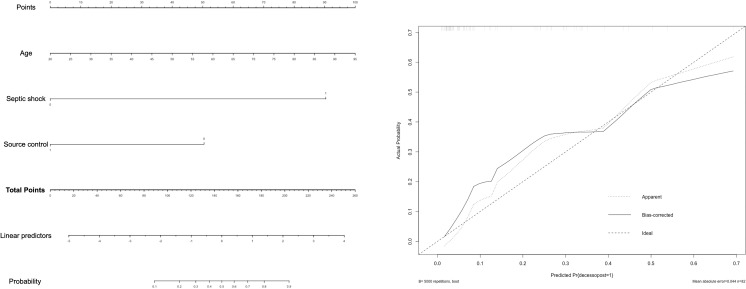
Multivariable model (A) and calibration curve (B) to predict the risk of death in imw patients with PA-BSI.

Based on our findings, a multivariable model to predict mortality among patients with PA-BSI in IMW was developed and internally validated (**Fig 2A**). A multivariable logistic regression model was adopted to choose the variables that best predict the occurrence of 30-day mortality. Within the nomogram (**Fig 2A**) each variable is visually represented with a corresponding score. The probability of death in patients with PA-BSI in IMW can be calculated by summing the points for each variable according to patient characteristics. This plot shows the predicted probability on the x-axis and the observed outcomes on the y-axis. We tested the prediction tool using calibration curves and assessed its performance. The calibration curve demonstrated a good consistency in predicting the risk of death when incorporating age, septic shock and source control. The discrimination ability of the model was measured using the optimism-corrected C-index, which stood at 78.57%. The calibration curve of the nomogram is illustrated in **Fig 2B**.

## Discussion

Patients admitted to IMW are often affected by multiple chronic illnesses requiring long hospital stays, invasive devices as central venous lines, urinary catheter or requiring immunosuppressive therapies [15]. Nearly 40% of BSI occur in IMW, and there is an increasing isolation of Gram-negative bacilli, including *P. aeruginosa* [16–20]. So far, data have described risk factors and PA-BSI related mortality mostly in the ICU setting and hematological patients, where the mortality rate is up to 30–35% [21].

To the best of our knowledge, this is the first largest multi-center retrospective study on clinical characteristics and risk factors for mortality of PA-BSI in IMW.

In our study, patients were elderly, with a median age of 73 years old, presented with several comorbidities and a low rate of hematological or solid cancer disorders. Of note, 20% of them had a central venous line, which was the most common origin of PA-BSI, unlike the ICU setting where respiratory tract is a common source of infections [22].

In the setting of CVC or urinary tract related bacteremia, source control seems easier to perform compared to others site of infection, but in our study, it was done only in 28.4% of patients. Moreover, adequate source control was protective for 30-days mortality at multivariate analysis, highlighting the relevance of an early identification of the source of infection and the need of a prompt removal to improve clinical outcome in the setting of PABSI.

We report here a low rate of septic shock at time of presentation (13.3%). This finding was in contrast to those reported in the literature: a large multicenter, retrospective cohort study including onco-hematological neutropenic patients with PA-BSI found that 33% presented with septic shock and combination therapy was frequently associated with reduced mortality. Similar findings have been described in an Italian monocentric study of PA-BSI, with a rate of 15% of septic shock and a beneficial influence of empirical double-active combination therapy on mortality in patients with neutropenia [21,23].

It should be noted that in both cited studies, patients were primarily admitted from oncology-hematology and ICU, that could explain the differences in the reported percentages of septic shock as well as the impact of combination therapy, which in our study did not prove to be significant in terms of mortality. Moreover our study had a low rate of neutropenic patients and steroids treatment, in which combination therapy may have a stronger impact on mortality as described in literature [21,22]. Of note, despite the Italian epidemiological setting with a high rate of carbapenem resistant bacteria spreading across the country, we report a rate of DTR PA lower than 5% [24–26]. This low rate of DTR infections might explain why most empiric therapies were appropriate, using either monotherapy or combination mostly with anti-pseudomonas βlactam or carbapenems. The statistical significance for 30-days mortality of target only therapy, administered in a small number of patients, should be interpreted considering the high rate of appropriate empiric treatment combined with a low rate of DTR infections. It should be noted that despite the low rate of DTR and cases of septic shock, we observed a 22.5% of 30 days mortality, similar to previous findings outside the ICU setting [22]. Multivariate analysis showed that septic shock was associated with a higher risk of mortality whilst adequate source control was protective. Therefore, our data suggest the need of improving source control when feasible, and underline the importance of early identification of patients with PA BSI in IMW at high-risk for mortality in order to choose the proper antibiotic therapy, which may include optimization of extended infusion or loading doses when piperacillin/tazobactam or carbapenems are deemed appropriate based on culture results, as well as the early use of new anti-pseudomonal agents for high-risk patients.

According to this, we identified a set of variables that combined could help to predict the likelihood of death in patients with PA BSI in IMW (**Fig. 2A**). Within the nomogram, each variable is visually represented with a corresponding score. The probability of death in patients with PA-BSI can be calculated in the nomogram by summing the points corresponding to each patient characteristic. A multivariate logistic regression model was adopted to choose the variable which better predicts the occurrence of 30-day mortality. For instance: a 70-yo patient, with suspected CVC related infection, without septic shock, than the corresponding score of this patient was 66 + 0 + 51 (total of 117 points) equivalent to a risk of death approximately 22%. The same patients with septic shock would have 66 + 87 + 51 points, raising the risk of death to over 90%.

Previous studies have established nomogram models to predict survival of patients with gram negative infections, including PA. However, it is worth noting that these studies were performed in intensive settings such as ICU or burn units [27,28] and not specifically performed in IMW. Previous studies reported increased organ failure, sepsis, and mortality rates in patients aged ≥65 with Gram-negative BSIs compared to younger patients. Also, patients aged ≥65 are more likely to present organ failure, septic shock, and longer hospital stays [29–31]. For this reason, although age was associated with mortality only in the univariate analysis of our study, it was selected for inclusion in the nomogram after calibration.

By using this bedside scoring system, physicians can quickly identify patients with PA-BSI who are at higher risk of death, and this could better guide their decision-making process in this frail population. Although further external validation trials are needed, preliminary calibration measures provided encouraging results. According to the predicted results, the scoring system could even be used to allocate new BL/BLI to patients who need them most as well as to spare carbapenems when feasible in patients with low-risk mortality.

Our study has several limitations. Although multicenter, this was a retrospective study, moreover the low rate of DTR-PA in our study limits the reproducibility of these data in settings with high prevalence of MDR organisms. Second, we built a nomogram to predict mortality in patients with PA BSI in IMW, but the nomogram needs further external validation to verify its performance. Third the low rate of DTR and the high rate of appropriate empiric therapies need to be considered when we evaluate the impact of appropriate therapy on 30-day mortality that needs to be further investigated.

## Conclusion

In conclusion, this is the first multicenter study assessing the burden of PA BSI in IMW. In our study, despite the low prevalence of DTR-PA and the high rate of appropriate empirical therapy, we unexpectedly observed a 22% mortality rate. Septic shock was significantly associated with 30-day mortality, whilst source control was strongly associated with survival. To improve outcomes in the IMW setting, it may be beneficial to promote source control through the removal of CVCs or urinary catheters, which was performed in only 28% of cases. When source control is not feasible, or the patient is at high-risk for mortality, treatment optimization through extended infusion or loading doses should be implemented when piperacillin/tazobactam or carbapenems are deemed appropriate based on culture results. Early administration of novel antipseudomonal agents should be considered in patients at high-risk for mortality, even if non-critically ill. Finally, we built a model to predict 30-day mortality in patients with PA-BSI. Therapeutic strategies that address the variables considered in this nomogram may help clinicians to modulate treatment choices when treating PA-BSI in IMW patients.
